# Beyond Plants: The Ultra-Processing of Global Diets Is Harming the Health of People, Places, and Planet

**DOI:** 10.3390/ijerph20156461

**Published:** 2023-07-27

**Authors:** Susan L. Prescott, Christopher R. D’Adamo, Kathleen F. Holton, Selena Ortiz, Nina Overby, Alan C. Logan

**Affiliations:** 1 Nova Institute for Health, Baltimore, MD 21231, USA; cdadamo@som.umaryland.edu (C.R.D.); alanxlogan@gmail.com (A.C.L.); 2 Department of Family and Community Medicine, University of Maryland School of Medicine, Baltimore, MD 21201, USA; 3 Medical School, University of Western Australia, Nedlands, WA 6009, Australia; 4 The ORIGINS Project, Telethon Kids Institute, Nedlands, WA 6009, Australia; 5 Departments of Health Studies and Neuroscience, Center for Neuroscience and Behavior, American University, Washington, DC 20016, USA; holton@american.edu; 6 Department of Health Policy and Administration, The Pennsylvania State University, State College, PA 16802, USA; suo13@psu.edu; 7 Department of Nutrition and Public Health, Centre for Lifecourse Nutrition, University of Agder, 4630 Kristiansand, Norway; nina.c.overby@uia.no

**Keywords:** ultra-processed foods, non-communicable diseases, public health, environmental health, sustainability, planetary health, mental health, commercial determinants of health, dietary excitotoxins, developmental origins of health and disease (DOHaD), social justice, climate change

## Abstract

Global food systems are a central issue for personal and planetary health in the Anthropocene. One aspect of major concern is the dramatic global spread of ultra-processed convenience foods in the last 75 years, which is linked with the rising human burden of disease and growing sustainability and environmental health challenges. However, there are also calls to radically transform global food systems, from animal to plant-derived protein sources, which may have unintended consequences. Commercial entities have moved toward this “great plant transition” with vigor. Whether motivated by profit or genuine environmental concern, this effort has facilitated the emergence of novel ultra-processed “plant-based” commercial products devoid of nutrients and fiber, and sometimes inclusive of high sugar, industrial fats, and synthetic additives. These and other ingredients combined into “plant-based” foods are often assumed to be healthy and lower in calorie content. However, the available evidence indicates that many of these products can potentially compromise health at all scales—of people, places, and planet. In this viewpoint, we summarize and reflect on the evidence and discussions presented at the Nova Network planetary health meeting on the “Future of Food”, which had a particular focus on the encroachment of ultra-processed foods into the global food supply, including the plant-sourced animal protein alternatives (and the collective of ingredients therein) that are finding their way into global fast-food chains. We contend that while there has been much uncritical media attention given to the environmental impact of protein and macronutrient sources—meat vs. novel soy/pea protein burgers, etc.—the impact of the heavy industrial processing on both human and environmental health is significant but often overlooked, including effects on cognition and mental health. This calls for a more nuanced discourse that considers these complexities and refocuses priorities and value systems towards mutualistic solutions, with co-benefits for individuals, local communities, and global ecology.

## 1. Introduction

“*In our time, educated consumers need to make food choices that not only enhance their own health but also contribute to the protection of our natural resources*.”

Drs. Joan Dye Gussow and Katherine L. Clancy, 1986 [1].

The concept of planetary health emphasizes that the health of human civilization is inseparable from the natural systems within the Earth’s biosphere. It recognizes the interconnectedness of health at different scales of “people, places, and planet”—encompassing individual well-being, local environments, communities, public health, and the overall health of interconnected systems ranging from the microbial to macroecological levels [2,3,4,5]. Planetary health seeks to understand these intricate connections by transcending artificial divisions between biological, psychological, social, and cultural aspects of health in the modern environment [6,7]. This is fundamental to addressing what has been termed “Anthropocene Syndrome”—the many wicked interrelated challenges of our time. These include, but are not limited to, grotesque biodiversity losses, climate change, environmental degradation, resource depletion, the global burden of non-communicable diseases (NCDs), social injustice/inequity, community breakdown, health, disparities, and unacceptable poverty of both income and opportunity [7,8].

One issue that permeates virtually all aspects of this discourse is food and nutrition—ranging from personal dietary patterns, food environments in local communities, and far-reaching global food systems [8,9,10,11,12,13,14]. This was the subject of discussion at our Nova Network planetary health meeting on the “Future of Food” held in March 2023 [15]. A central theme was on the dramatic global increase in ultra-processed food production and consumption since the 1950′s and evidence that this is linked with the rising burden of both human disease and environmental degradation with transgenerational implications.

**Figure 1:**
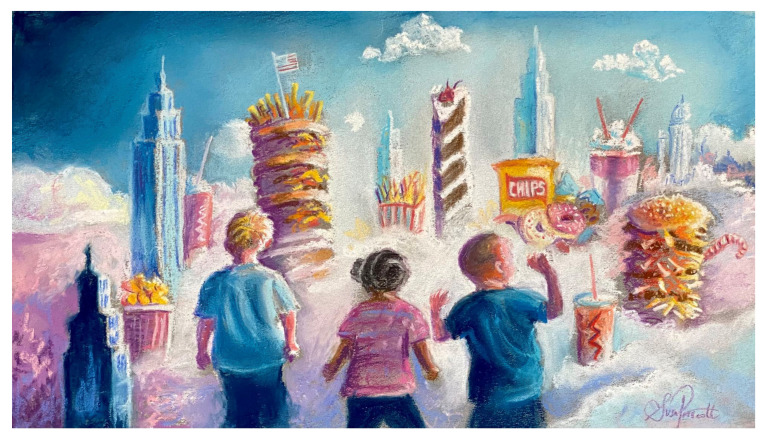
Unhealthy food systems are implicated in the rise in many NCDs through early-life effects. Dietary patterns have a profound influence on structures, physiological responses, taste preferences, eating behaviors, and the microbial ecosystems critical to our life’s long-term physical and mental health. Artwork created by author S.L.P.

Like other environmental exposures, nutritional patterns during critical stages of development have profound effects on the structures, functions, and developing behaviors that contribute to health span and lifespan [16]. This includes subsequent adult food preferences and eating behaviors that contribute to the lifelong risk of NCDs. Food marketing to young children is linked to significant, negative effects on food preferences, choices, and consumption [17,18], with major long-term implications for the global NCD burden (Figure 1). Furthermore, adverse exposures, especially nutritional, can also have heritable epigenetic changes that transmit increased risk of disease to second and even third generations [19]. Our discussions were therefore framed around the imperative of taking long-term, life-course perspectives on disease prevention that also considers the wider social, economic, and commercial determinants of health [20].

We also explored how ultra-processed food technology has been utilized in the “great plant transition”—calls to move from animal to plant-derived protein sources as part of “planetary diets” [21,22]. While this transition is well-intended to reduce the ecological footprint and health burden of high animal protein consumption, the emergence of ultra-processed plant-based products as a central part of this strategy may have unintended consequences for both human and environmental health.

Here in this viewpoint, we synthesize, summarize, and reflect on the proceedings of this meeting, in the context of the literature. We begin by discussing the historical aspects of diets promoted for both personal and planetary health and the coincident global spread of convenience foods subjected to high levels of industrial processing. This includes the broadly defined term “plant-based diet,” how this is conflated with evidence-supported health-promoting diets, and the ways in which multinational corporations leverage the term to justify a wide variety of ultra-processed commercial products. As we discuss, many of these “plant-based” products are devoid of nutrients and fiber and inclusive of high sugar, industrial fats, and large amounts of additives. It is our contention that the sense of urgency to “radically transform” global food systems [21,22] for the “great protein transition” to plant-derived sources [22] has unwittingly facilitated the emergence of ultra-processed foods and novel commercial products that include ingredients that may compromise health. Considering the emerging evidence that these ultra-processed food dietary patterns (including macronutrients, micronutrients, and dietary additives) impact neuropsychiatric outcomes [23,24,25,26,27,28,29,30,31,32,33,34], we approach our discussion from the perspective of mental health. At the outset, we underscore that throughout human history, various forms of food processing have been an essential contributor to human welfare, and there is little doubt that solutions to the problems of over/undernutrition, within the context of sustainability, will involve the expertise and cooperation of the food industry [35,36].

## 2. Historical Aspects of Planetary Health Diets

From its early origins in the 1970s and 1980s, the planetary health movement emphasized a diet rich in relatively unprocessed fruits, vegetables, and grains, and minimal amounts of meat, as a way to promote health along the continuum of persons, places, and planet [6]. In the early 1990s, popular press writers were promoting the idea that heart-healthy diets (lower in meat) also reduce demand for various resources, including fossil fuels: “*the American penchant for these dietary no-no’s* [excess meat] *is also bad for our planetary health*” [37]. At the time, the National Cattlemen’s Association dismissed the literature linking heart-and-planet healthy diets, with spokespersons informing the media that “*everyone has a how-to-save-the-world book these days, and they’ve regurgitated the same* [false] *information*” about needing to eat lower on the food chain [38].

Initial exploration of planetary health diets included various versions of the “macrobiotic” diet, a plant-based diet with minimal processing, limited consumption of added sugars and dietary additives, and a general avoidance of meat and animal-derived foods. Despite its well-intended claim to be *the* diet for planetary health [39], this was considered faddish [40]. With its roots in traditional Asian medicine (a diet said to balance yin/yang), western scientists referred to the diet as ‘quasi-religious’ [41]. In the 1980–1990s, researchers found that strict adherence to the macrobiotic diet caused harm, as it was associated with various nutritional deficiencies such as prolonged vitamin B12 deficiency [42] and reduced bone mass [43] in adolescents who were fed a macrobiotic diet in early life, as well as delays in childhood neurocognitive development [44,45]. With the inclusion of nutrient fortification and awareness of supplementation options, variants of the macrobiotic diet have increased in popularity, and recent studies show that the basic principles of the diet have potential health benefits [46,47,48,49] despite its drawbacks. Indeed, the basic principles of the macrobiotic diet—whole grains, colorful fruits and vegetables, and legumes—are generally in line with the global reference diet (also known as the “planetary health diet”) published more recently by the EAT-Lancet Commission on Healthy Diets from Sustainable Food Systems (EAT-Lancet) [50]. The EAT-Lancet diet recommends minimal red meat; the upper limit of daily red meat intake is about five times less than that found in *healthy* Mediterranean and United States patterns [51], and almost eight times less than the current daily intake of meat among US adults [52]. 

It should be noted that the causal evidence linking unprocessed red meat to non-communicable disease mortality is reportedly weak [53] and adherence to the EAT-Lancet diet has not, thus far, been linked to reduced mortality from cardiovascular disease and cancer [54]. In fact, it has been argued that the EAT-Lancet diet may contribute to the increasing burden of mental illness [55] and that the guidance offered is not feasible [56,57] on a global basis and may exacerbate food inequity. Meat consumption has been linked to lower rates of depression and anxiety, which may be reflective of select nutrients and amino acids within the whole food [58,59,60,61]. Nevertheless, there is a general agreement that a diet rich in whole fruits, vegetables, and fiber-rich grains, with lowered meat intake from industrial feedlots (and especially reduced consumption of processed red meat from these sources), is in the interest of health promotion at scales of people, place, and planet. Based on recent trends, the profit-driven response to planetary health (vis à vis food systems) appears to place less emphasis on the health of individual people. Specifically, we argue that the vague but merit-loaded term “plant-based diet” has allowed for the spread of ultra-processed “plant” foods and the rapid emergence of additive-rich “biomimicry burgers” and other meat alternatives. The extent to which these products, including cultured meat, reduce the environmental burden remains an open question [62].

## 3. The Rise and Rise of Ultra-Processed Foods

The global spread of ultra-processed foods has risen in tandem with economic development and urbanization [63]. It is difficult to know with certainty when and in what context the term ultra-processed food emerged. There is little question that the rise of highly processed convenience foods (referred to as “ultra-refined” foods) in post-World War II America was already causing alarm among physicians by 1950 [64,65,66]. Over fifty years ago, the massive influx of “ultra-processed flour” (grain flours stripped of fiber and nutrients), especially when combined with added refined sugar, was already linked to high palatability, excess caloric intake, obesity, and colon cancer [67,68,69]. In the 1970–1980s, the terms “ultra-processed” and “ultra-processing” were used internally within food industry journals and literature in reference to new product developments, especially those extending shelf-life and convenience, ranging from spray-can whip cream [70] to pourable liquid eggs [71]. In a 1982 *Esquire* article entitled *The Universal Salesman*, author Laurence Shames took exception to the dietary “disparity” of marketing executives who peddled “*ultra-processed foodstuffs whose labels read like a chem text*” while consuming minimally-processed whole foods in their own lives [72]. By the end of the 1980s, the term ultra-processed foods had entered the public lexicon through pop health books that referred to the rise and dominance of highly processed foods as the “era of ultra-processed foods” [73,74]. In 2000, newspaper editorials opined that the low-fat diet fad was giving rise to “package upon package of ultra-processed, high-carbohydrate foods of every variety” [75].

Despite the increasing use of the term ultra-processed foods, it was much like the term plant-based diet, lacking specificity and holding very little scientific value. Beginning in 2009–2010, “processed” foods were classified in the context of overall food processing, with the understanding that much of food industry processing provides tremendous value to personal and public health at local and global scales. At issue was the extent to which important food processing techniques (such as removing inedible fractions, grating, squeezing, draining, flaking, drying, parboiling, fermentation, pasteurization, pressing, crushing, milling, etc.) are subjected to additional processing (and often inclusive of higher levels of sugar, fat, emulsifiers, flavor enhancers, synthetic ingredients, plant isolates, and extruded meat remnants) to create durable, accessible, convenient, and palatable foods/drinks that are mostly ready-to-eat or to-heat [76,77]. Monteiro and colleagues showed that it is possible to break down household food purchases into three major groups based on the degree of processing, with a distinct group referred to as ultra-processed foods [78,79,80]; this led to the development of what is now known as the four-category NOVA food classification system, including (i) unprocessed/minimally processed foods, (ii) processed culinary ingredients, (iii) processed food products, and (iv) ultra-processed products [81,82].

The introduction of the NOVA system was a significant advance over previous (less-specific) attempts [83] at assessing the extent to which highly processed foods had entered national food supplies. In particular, it is possible to capture the consumption of ultra-processed foods at national levels, and within subpopulations, including marginalized communities [84]. As discussed below, although increasingly utilized, the NOVA system is not universally accepted [85]. At present, ultra-processed foods are estimated to account for approximately 57–60% of daily energy intake in the US and UK [86,87] and 50% throughout Europe [88]. Unless there is a significant reversal in trends, the current large-scale encroachment of ultra-processed foods into the food supplies of low and middle-income countries is estimated to reach that of Westernized nations (≈60% daily energy intake) within the next few years [89].

The increasing share of ultra-processed foods in household food expenditures/energy intake is a significant personal and public health concern because intake of these foods has been linked to a higher risk of multiple diseases and disorders, both infectious and non-communicable, and excess mortality [90,91,92,93,94,95]. In addition, a higher intake of ultra-processed foods has been linked to psychological distress, sleep disturbances, and mental disorders, including depression and anxiety [23,24,25,26,96,97]. We will address the potential neuropsychiatric consequences of ultra-processed foods in more detail below. For now, in the context of planetary health, it is important to underscore that since many ultra-processed foods are “plant-based”; the wide distribution of such products deserves scrutiny from an environmental perspective.

The available research indicates that the global distribution and sales of ultra-processed foods (inclusive of foods generally described as “discretionary”) are at odds with the sustainability priorities of a planetary health diet [98,99,100]. Longitudinal research shows that reductions in ultra-processed food intake and more alignment with a Mediterranean-style dietary pattern are associated with a lowered environmental footprint [101]. Moreover, research has shown that ultra-processed food consumption (vs. ad libitum minimally processed food access/consumption) is associated with greater caloric intake and weight gain [102]. On the global scale, energy intake and industry/marketing-driven obesity [103,104] add to a significant environmental burden, including over 20% higher greenhouse gas emissions, water use, and land use [105,106]. Associations between obesity and environmental burdens (especially fossil fuel consumption) have been in discussion since the 1970s [107]. However, historical discussions of obesity have emphasized personal responsibility, stigmatized individuals, and allowed its upstream drivers (e.g., multinational corporations engaging in target marketing [108,109,110] of vulnerable and marginalized communities; poverty; food insecurity; and precariousness) to escape scrutiny [111]. At the same time, terms such as “metabolically-healthy obesity” infer that obesity is benign, and this, too, favors the narrative of large multinational corporations pushing ultra-processed foods under the “healthy-at-any-weight” banner; available evidence indicates that “metabolically-healthy obesity” is not benign [112,113]. Indeed, relationships between excess weight and mortality have likely been underestimated [114]. At the same time, it is important to point out that associations between ultra-processed foods (including their primary ingredients, discussed below) and non-communicable diseases are not dependent upon overweight and obesity [115,116].

While estimates of the global environmental costs of obesity, as described above, have focused on agricultural production phases and transportation, the burden of obesity on the energy consumption and greenhouse gas production of healthcare systems remains untold. Taken as a whole, the healthcare system in the US has an outsized environmental footprint [117], and it is highly likely that the healthcare-associated footprints of low/middle-income countries will grow in concert with the dietary expansion of ultra-processed foods. Brazil, a transitioning middle-income country with increasing obesity rates [118], provides an example: ultra-processed food consumption as a percent of daily energy intake is rising, although it remains at present less than half of that in the US and Europe. Nevertheless, ultra-processed foods are already placing a significant burden on healthcare systems and are currently responsible for an estimated 10.5% of all premature deaths in adults aged 30–69 years [119].

## 4. Sugar and Fat

The NOVA classification has been criticized for its “all-inclusive” approach to a collective of dietary components that have already been connected to diseases and disorders. That is, the NOVA system, according to critics, is merely capturing sugar intake, excess saturated and omega-6 polyunsaturated fat intake, and overall unhealthy dietary patterns. Excess dietary sugar, for example, has emerged as a stand-alone risk factor for multiple non-communicable diseases [120,121,122] and has been linked to depression and other neuropsychiatric disorders [123,124,125]. Of critical importance is the emerging research demonstrating that dietary choices can have transgenerational influences on behavior, mental health, and even aspects of personality; for example, maternal and early childhood dietary patterns that are healthy and sustainable predict benevolence, conscientiousness, and imagination (and lower neuroticism) at age eight [126]. This research underscores the developmental origins of the health and disease perspective, indicating that early-life experiences with sugar and ultra-processed foods are programming later health risks [127,128].

There is little question that ultra-processed foods are often determinants of added dietary sugars, and the rise of ultra-processed foods has been noted to be in parallel with added sugars [129]. The ultra-processed food dietary pattern has also been found to be characteristically high in omega-6 (vs. omega-3) fatty acids [102], and omega-6 dominance has been linked to psychological distress, depression, and other mental disorders [130,131,132]. In the longitudinal Moli-sani Italian study, researchers reported that high sugar intake mediates 36.3% of the relation between ultra-processed food with ischemic heart disease/cerebrovascular mortality [133]. The NOVA system has also been challenged as a mere surrogate marker for overall nutrient intake, including fiber, or lack thereof [134,135]. Dietary patterns high in ultra-processed foods are typically lower in phytochemical antioxidants such as polyphenols [136,137]. However, despite challenges to the NOVA system, much of which has been from individuals and groups with strong industry ties [138,139], the available evidence shows that the majority of the associations between ultra-processed food intake and obesity, and other health-related outcomes, remain significant after adjustment for dietary contents of critical nutrients and dietary patterns that take into account critical nutrients and food groups [140]. Still, the NOVA food classification system cannot always distinguish between healthy and unhealthy foods [141], and a lack of consistency among food evaluators using NOVA can be problematic [142]. 

## 5. Wide-Ranging Health Effects: From Excitotoxins to Mental Health

**Figure 2:**
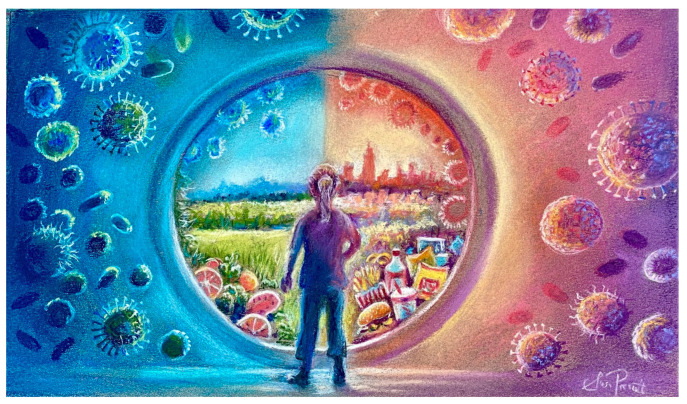
The dramatic rise in non-communicable diseases (NCDs) with progressive urbanization is associated with changing gut microbiomes and predisposition to chronic inflammation. While this is multifactorial, western dietary patterns, including UPF, are one of the major factors implicated in this microbiome-associated metabolic and immune dysregulation. Artwork created by author S.L.P.

Although less attention has been paid to the dietary additives captured in the NOVA classification, volumes of pre-clinical research have linked the non-nutritive components of foods (e.g., emulsifiers, flavor enhancers, and artificial colors) to non-communicable diseases, including gastrointestinal and mental disorders [143,144,145,146,147,148,149,150]. In animal models, various dietary additives, including emulsifiers and monosodium glutamate (MSG), have been shown to alter the gastrointestinal microbiome [151,152,153,154]; ultra-processed food-induced alterations to the gut microbiome might account for many of the diet’s health-related consequences [155] (Figure 2). Indeed, compared to either MSG or a high-fat diet alone, the combination of MSG and a high-fat diet (i.e., characteristic of ultra-processed foods) has been noted to cause a shift in gut microbiota reflective of an obesity signature [156]. The absence of dietary nutrients such as antioxidants and omega-3 fatty acids might worsen the ADHD-like behavioral changes of animals exposed to MSG [148,157,158]. We will discuss MSG in more detail below. For now, it is important to note that these pre-clinical studies provide a mechanistic understanding of the observed negative synergy between flavor enhancers and the composition of many ultra-processed foods [156,159].

The consumption of foods and beverages containing dietary additives continues to grow in the US; perhaps most concerning is a reported 20% increase in the proportion of baby food purchases containing additives and an over 15% increase in the proportion of purchases containing three or more dietary additives [160]. Dietary additives are included in the majority of foods in Europe, Australia, and Brazil [161,162,163]. However, it is important to note that based on publicly available government databases, the United States far outstrips Canada and countries in the European Union in the number of additives that have been approved for use in food (e.g., thousands of approved additives versus hundreds) [161,164,165]. Although much of the research on dietary additives such as emulsifiers and flavor enhancers remains in pre-clinical animal models, epidemiological research has linked MSG and obesity/metabolic syndrome [166,167] and dietary emulsifier intake with Crohn’s disease and ulcerative colitis [168,169]. Researchers in the field of nutritional neuroscience have recently extended McCann’s controlled study in the *Lancet* [170] linking artificial (dietary) colors with changes to brainwave activity and neuropsychiatric symptoms in adults with ADHD [171].

Glutamate (the main component in MSG) and associated “dietary excitotoxin” chemicals such as aspartate [172] are worthy of detailed examination within the ultra-processed food discourse. These chemicals, referred to as excitotoxins because of their capacity to overexcite neurons, have been associated with pain sensitivity and neuropsychiatric symptoms; and it has been hypothesized that differences in blood-brain barrier permeability in various non-communicable diseases might determine the presence/absence of dietary excitotoxin symptomology [173,174]. Multiple pre-clinical studies show that MSG has the potential to have deleterious effects on nervous symptom function, including heightened pain sensitivity, and alterations to animal behaviors that reflect human depression and/or anxiety [175,176,177].

While there is little doubt that MSG enhances food palatability in human subjects [178,179], there has been surprisingly little controlled human research on the ‘after-effects’ (neuropsychiatric sequalae) of MSG-inclusive foods in the post-prandial phase and the 24 h post-consumption phase. However, recent human studies have investigated the potential health benefits of dietary interventions emphasizing the elimination/low intake of excitotoxin additives such as MSG and aspartame; the results to date have noted improvement in PTSD [180,181], fibromyalgia [182], and pain sensitivity and cognitive function as well as depression, anxiety, and PTSD [181] in veterans with Gulf War Illness [183,184,185]. Questions concerning the post-prandial biopsychological consequences of dietary excitotoxin-rich foods are related to the paradox of ‘comfort foods’— although there are acute post-consumption perceived benefits of highly palatable ultra-processed foods (i.e., brief mood elevation and reduction in stress), the long-term consumption places a physiological burden on the body [186]. The extent to which individuals can trace various neuropsychiatric signs and symptoms back to prior consumption of dietary excitotoxins, especially when they are embedded in multi-ingredient ultra-processed foods, is an open question. The extent to which the dietary additives with ultra-processed foods might be independently contributing to diseases and disorders, and whether or not the preclinical work fully extends to humans, is an ongoing area of research.

## 6. Dietary Displacement, Planetary Health

“*In many cases when new foods are introduced it will be very difficult to forecast exactly where substitution will occur. If we introduce ‘vegetable-protein steaks,’ will they replace real steak, or will they replace sausages or even bread?*”

BRW Pinsent, Unilever food scientist, 1970 [83]

Taken as a whole, the available evidence indicates that the growing encroachment of ultra-processed foods into the global food supply is potentially harmful at scales of persons, places, and planet. Although there has been increasing media attention and public awareness of the potential harms of ultra-processed foods, the extent to which the industry has leveraged the ‘radical need’ for dietary transitions to a plant-based diet has received little scrutiny. Recent years have witnessed large-scale new product developments and marketing of “plant burgers” and other alternatives to animal protein sources. These are ultra-processed foods, often inclusive of many of the problematic ingredients described above.

The quote above, from a Unilever food chemist, highlights that multinational corporations were well aware, even 50 years ago, that the development of plant-derived meat alternatives could displace healthy nutritional choices and might not even replace meat in the overall diet. While this might sound far-fetched, consider that Burger King has recently added multiple strips of bacon as an option on top of its ‘plant-based’ Impossible Burger [187]; sales of the Impossible Burger have been flagging since the initial product launch and associated media attention [188]. Research has shown that those who avoid meat consumption are more likely to consume ultra-processed foods, especially among younger adults and those who are recent adopters of a vegetarian or vegan diet [189]. Diets with novel plant-based alternative or substitute foods have been reported to lack the daily requirements for calcium, potassium, magnesium, zinc, and vitamin B12, while containing excess saturated fat, sodium, and sugar [190]. Mass-market, fast-food, ‘plant-based’ burgers contain significant amounts of sodium [191] and the processed protein sources in the foods, as well as other additives in these products, can also be sources of dietary excitotoxins, which could negatively affect sensitive individuals.

The basis of many plant-based meat alternatives is highly processed soy protein isolate, which is rich in isoflavones (i.e., secondary metabolites belonging to the phytoestrogen family). Isoflavones, which have chemical structures similar to 17β-estradiol [192], are capable of binding to estrogen receptors and have been shown to have adverse impacts, “*the unknown consumption of doses and types of isoflavones in food can damage the development and reproduction of individuals*”, according to an expert review [193]. The large influx of isoflavone-rich soy isolate (distinct from traditional diet whole foods, fermented soy culinary items) into the global dietary supply may have untold developmental consequences and, especially in males, influence mental health [194,195,196]. It is worth pointing out that textured soy protein, which is added in large amounts to animal meat products as an “extender” [197], contains eight times more isoflavones than traditional soy milk [198]. Many consumers might be surprised to know that supermarket “beef patties” can contain as much as 30% textured soy protein [199,200]. The consumption of hidden isoflavones (i.e., in products not overtly recognized as soy products like tofu, soy milk, etc.) is an area of increasing concern [201]. Some authors, especially those with direct soybean industry ties (e.g., the “Soy Nutrition Institute Global”, Cargill Inc., etc.), propose that soybean-based meat alternatives should not be considered ultra-processed foods [202] and that soybean isoflavones should not be classified as endocrine disruptors [203]. The extent to which the pre-clinical endocrine-disrupting effects of soy isoflavones (and their gut microbiome metabolites) extend to humans remains an open question [204]. The extent to which the soy industry is shaping international discourse is also an open question [85,205,206].

Related to the topic of soy is the marked expansion of the plant-based beverage industry, especially those that are intended to act as a substitute for milk and other dairy-based drinks. While soy, oat, almond, and rice, beverages were once a niche health food store market, the 2022 sales of these products exceeded US $3.1 billion dollars in the United States and US $20.9 billion globally [207]. Although at first glance such beverages appear to be superior to dairy milk in terms of environmental impact, such might not be the case when nutritional factors (protein and various nutrients) are brought into the assessment [208,209,210]. Almond milk, the number one dairy beverage alternative in the US, utilizes significant quantities of water during manufacturing [211]. The nutrient shortfalls of milk alternatives have been well-described [212]. Moreover, even when fortified with protein and calcium to match dairy milk, many of these milks contain significant quantities of sugar, emulsifiers, and other additives [213,214]

The plant-based meat discourse needs to extend beyond nutrients per se and into the realm of processing, a topic currently avoided by purveyors of meat alternatives [215]. The findings above suggest that consumers of plant-based substitutes are also more likely to be exposed to the food additives (emulsifiers and excitotoxins) that are common to ultra-processed foods. Meat substitutes, such as fast-food chain “plant-based” burgers, often include ‘yeast extract’ (flavor enhancer, a vague term often used for glutamate-rich MSG-like excitotoxins [216]) and the emulsifier methylcellulose (linked to gastrointestinal inflammation [217]). In experimental models, plant-based alternative burgers (vs. meat) raise the *Firmicutes* to *Bacteroidetes* ratio in gut microbiota [218]. Although there have been disparate findings, the preponderance of studies has linked a higher *Firmicutes* to *Bacteroidetes* ratio to irritable bowel syndrome and other non-communicable diseases [219,220,221]. Given the mass distribution of novel meat alternatives and the far-reaching influence of gut microbiota, including neuropsychiatric health, the microbiome is an area worthy of intense scrutiny.

## 7. Where to From Here?

Based on the unbridled rise of ultra-processed foods within the global food supply, there is an urgent need to dissect the policies and practices that have allowed such unhealthy products to dominate commercial landscapes and household purchasing. This includes a deeper understanding of the tactics used by multinational food/beverage corporations, many of which are derived from the marketing–lobbying playbook of tobacco companies [222,223]. This deeper analysis is part of the emerging concept known as the commercial determinants of health [111,224]. At the same time, there is an urgent need to develop policies and practices that encourage the consumption of minimally processed foods [36] and reward the many commercial entities that can aid in that process, especially those that adopt health-promoting practices, portfolios, resources, organizational structures, and transparency [225].

The monetary cost of ultra-processed foods varies according to geography [226]; in some locations, these foods are less expensive than healthy whole foods [135,227], although this is not always the case [228,229], and ultra-processed food intake isn’t always highest in groups with the lowest household income [230]. Several studies show that taxation and other price-raising policies directed at ultra-processed foods, in real-world settings and forecasting models, have the potential to lower intake [231,232,233] and associated non-communicable disease risk [234]. Other policy efforts include front-of-package warning labels, significant restrictions on advertising/marketing, and meaningful changes to school nutrition policies [235,236,237].

Experts have argued that combinations of these approaches may offer a synergy in reducing ultra-processed food consumption [238]. Yet, how to move the public toward food policies that support well-being at scales of person, place, and planet? Research shows that one method is to increase consumer awareness of the ways in which industry uses food engineering tactics to maximize palatability; included in this taste engineering frame are the combined physiological (e.g., blends of salt, sugar, fat, and flavor enhancers), cognitive (e.g., marketing/packaging and inaccurate perception of healthiness), and food environment (e.g., “value” meals, vending machines, shelf positioning, and point-of-purchase to leverage impulsivity) form of industry engineering. Framing messages that highlight the universality of industry engineering, the way it is used to manipulate the environment and tap into involuntary actions, and which knowingly create risk, increases support for food-industry-related obesity prevention policies [239]. In support of these findings, young people are more likely to make healthy dietary choices, and see healthy eating through the lens of social justice, when they are made aware of the manipulative practices of the food industry [240,241].

Given the calls for a radical transformation of the global food supply, and the ongoing ‘protein transition’ in particular, there is an urgent need for controlled studies to measure the impact of refined ingredients, both in isolated form and as part of ultra-processed foods, including meat substitutes [242,243]. At present, animal protein has been represented as an intrinsically harmful food in the context of planetary health, which has led to an assumption that ‘proteins’ are interchangeable and the level of processing and food matrix is of little importance [244,245]. Research shows that restaurant visitors *believe* that plant-based meat alternatives are healthier than meat and greatly underestimate the caloric content, fat, and sodium, in these products [246]; however, there is little scientific basis for the belief that plant-based meat alternatives are healthy (let alone healthier) choices.

Despite the manufacturer-described environmental benefits of plant burgers, nuggets, sausages, and what not [246], these items sold in fast-food restaurants might actually increase the real-world consumption of industrial feedlot meat; consider that the availability of healthy options in fast-food outlets, even though they make up a minimal number of sales, allows for visits by consumers with diverse tastes. Imagine a group of five co-workers, friends, or family, one of whom wants a salad or vegetarian/vegan option. Where will the group decide to eat? The availability of the “healthy” or vegetarian option allows all five of the group to visit the fast-food outlet—a phenomenon known as “veto-proof” decision-making [247,248]. Put simply, it is unknown whether plant burgers are increasing the sales of other meat-based products through the “veto-proof” group effect. Moreover, while messages concerning animal welfare and the personal and environmental health effects of reducing red meat consumption seem to work at full-service dining establishments, none of these messages influence red meat selection from quick-service outlets [249], indicating that the central problem lies with the fast-food outlets and all that they represent. These outlets are not being supplied by bucolic family farms prioritizing animal welfare [250]; in the US, a tiny minority of facilities, less than 6%, produce 89% of the animals and about 85% of the greenhouse gas emissions of all animal production [251]. It is important to note here that fast food consumption, as the major driver of feedlot beef production, must be reduced if we are to have a large positive impact on the environment. Encouraging people to not go to fast food establishments (including veggie burger alternatives) and to instead eat only organic grass-fed beef on a less-frequent basis may be an important driver of change that should be part of the discussion. We recognize, however, the multiple considerations that influence the purchase and consumption of fast food, such as perceptions regarding affordability and convenience, particularly among low-income populations. These, and other factors, should also be included in the discussion.

Moving forward, the development of policies and practices supportive of health at scales of person, places, and planet will require science-based journalism, a media effort that will deeply scrutinize the tactics of multinational corporations. Journalists continue to struggle with identifying multiple layers of conflicts of interest among featured experts [252,253]; part of this can be traced to industry nesting within the upper echelons of influential nutritional organizations [254,255]. At present, the media often promote the agenda of fast-food and other ultra-processed food purveyors; for example, when Burger King engaged in a 2019 marketing campaign, supposedly promoting mental health awareness by placing Whopper-Fries-and-a-drink meals in colorful mood-based boxes (so-called Real Meals) [256], top-level media ran promotional headlines such as “Burger King’s ‘Real Meals’ are about more than trolling McDonald’s. They’re about mental health” [257]. Media lauded McDonald’s for giving out free fast-food meals and placing vaccine messaging on its packaging during the COVID-19 pandemic [258,259], but elided the potential of ultra-processed foods (including fast-food) in contributing to COVID-related hospitalizations and mortality [91,260,261]. What is required is a media that understands the far-reaching, transgenerational implications of the nutritional psychiatry research [126], with a willingness to query the corporation’s role in potentially contributing to depression, anxiety, and other mental disorders; moreover, there is a need to consistently discuss these factors in the person, place, and planet context, rather than in isolation [11].

## 8. Conclusions

It is often said that we cannot have healthy humans on a sick planet. The reverse option of sick humans on a healthy planet is not a desirable goal. Our focus here is on mental health in the context of the person, place, and planetary health continuum; the World Health Organization position that there is ‘no health without mental health’ [262] provides an excellent guidepost toward next steps and future considerations. Currently, the *direct* healthcare costs of mental disorders exceed USD $200 billion [263], a figure that is likely a mere fraction of the trillions of global expenditures related to mental health (including substance use, often neglected from financial figures) as it intersects with poverty, racism, criminal justice systems, comorbidities, transgenerational inequities, and environmental injustices [20,264]. Put simply, when querying current policies and practices with an eye toward sustainability, we must ask whether existing and/or planned nutritional policies are in the interest of mental health, and that includes the oft-described ‘radical changes’ to the global food supply. Based on the available research, the rise of ultra-processed plant foods, including nouveau plant-based meats served up by fast-food chains, is not in the interest of mental health. Isoflavone-rich, emulsifier-, and excitotoxin-inclusive plant-based burgers, topped with four slices of bacon, look less like a planetary health diet meal and more like “low tar” cigarettes.

## Data Availability

Not applicable.
